# On the Enhanced Accuracy of Kinetic Curve Building in Supercritical Fluid Extraction from Aroma Plants Using a New 3D-Printed Extract Collection Device

**DOI:** 10.3390/molecules25092008

**Published:** 2020-04-25

**Authors:** Denis Prokopchuk, Oleg Pokrovskiy

**Affiliations:** Kurnakov Institute of General and Inorganic Chemistry of Russian Academy of Sciences, Moscow 119991, Russia

**Keywords:** supercritical fluid extraction, kinetic curve, collection accuracy

## Abstract

Accurate collection of extracted material represents a technical problem in supercritical fluid extraction because trapping should be performed in severe conditions of rapidly moving and freezing expanded fluid. We have developed a simple device for effective sample collection in analytical-scale supercritical fluid extraction. The device consists of a cyclone separator equipped with a spray trap and a heated check valve. The cyclone separator and spray trap are manufactured from a light polymer via 3D printing and are quick-detachable, which encourages its use in applications where mass yield measurements are required. The device was compared to a standard tubing-and-vial approach in the task of building kinetic curves for the extraction from two aroma plants, namely, laurel and rosemary. The new device showed almost two-fold increase in extraction trapping, most probably due to better collection of volatile compounds. A curious effect of the number of mass measurement points per curve on apparent yield was observed. An increase in the number of points led to an increase in yield, probably due to the effect of the static–dynamic extract regime posed by the manner in which the device is used.

## 1. Introduction

Liquid extraction is one of the major techniques for the isolation of chemical compounds, both from solid and liquid samples. Modern extraction systems focus on treating complex matrices in an easy, cost-effective, and ecologically friendly way [1,2,3,4,5,6,7]. On-line sample pretreatment brings several additional advantages to extraction technology, such as higher speed, lesser solvent use, and better process control [5,6,7].

Supercritical fluid extraction (SFE) is already a well-established, yet still relatively new, method of low-to-medium polarity compound isolation from various matrices. It is used in both analytical work as a sample preparation technique [8,9,10] and on a preparative scale for the isolation of valuable compounds from different matrices [11,12,13] as well as for cleaning porous materials [14] and precision devices [15], aerogel drying [16], etc. Method development in SFE is more complex than in traditional liquid extraction due to a broad spectrum of parameters affecting the results. One of the main features of SFE that drives its spread is automatic solvent evaporation after extraction, which allows solvent-free extracts to be obtained. This is important both on analytical and preparative scales as extracts are collected in a concentrated form and contain no solvent residues. However, this same beneficial feature also creates a serious problem during method development. Modern SFE is almost always performed in a flow manner. Supercritical CO_2_ coming out of the high-pressure system bearing an extract experiences a 100 times volume expansion, which leads to the formation of a high-speed flow of aerosol and to a severe decrease in temperature due to the Joule-Thomson effect. Both these factors hamper accurate extract collection, which is a key procedure during method development. One has to collect, preferably quantitatively, both droplets and vapors of extract components from a gaseous flow moving with high linear velocity and simultaneously avoid problems caused by adiabatic cooling, such as dry ice formation and tube clogging. On an industrial scale, these issues are solved by performing collection at elevated pressures in large-volume cyclone separators. On an analytical scale, large-volume collection vessels are not applicable, and other solutions should be employed.

This problem has been recognized by SFE enthusiasts for decades. There are basically four approaches for dealing with it: (i) online extract analysis, (ii) extract absorption by a proper solvent, (iii) extract adsorption onto a proper sorbent, and (iv) extract cryogenic entrapment.

The first approach is arguably the most effective one if the task in question is qualitative analysis of extract chemical composition. Large progress has been made in that direction recently. Particularly great success has been achieved in the field of online hyphenation of SFE and analytical supercritical fluid chromatography (SFC). Such a combination has always been recognized as a naturally blessed one due to close proximity of methods [17], and there has recently been a boost in efforts in that direction [18,19,20]. It has partly been stimulated by the appearance of commercial instruments providing the opportunity of performing online SFE–SFC; besides, further technical enhancements have been developed by experienced practitioners [21]. Other chromatographic techniques are, of course, hyphened to SFE as well [17,19,22]. For instance, tandem of SFE with gas chromatography (GC) is particularly promising in the field of aroma plant extract analysis, whereas liquid chromatography (LC) is more prudent when extracts contain mostly non-volatile components [19,22]. Chromatography-based online analysis methods are not the only ones applicable for SFE. Spectroscopic methods, such as IR, UV/Vis, evaporative light scattering detector (ELSD), etc. can be employed as well. If information on particular compounds is required, spectroscopic methods would require developing an approach for deconvolution and interpretation of signals from different compounds. Successful realization of this approach using both UV/Vis and ELSD was demonstrated recently [23,24,25].

Online analysis methods are excellent if one needs to monitor the extraction of major constituents. Unfortunately, they are useless when total extract mass quantification is required. Natural product extracts contain hundreds of chemical compounds of very diverse structure. No chromatography or spectroscopy technique, however elaborate, can quantify all of them in one run. In order to solve this task, one has to collect the sample and weigh it off-line. As stated above, this represents a problem in analytical-scale SFE. Entrapment of extract into a solvent [26,27,28] or onto a sorbent [29,30,31,32,33,34] are two preferable strategies for quantitative sample collection in SFE; when working with volatile extractables, they are frequently accompanied by cryogenic cooling [29,35,36,37]. Alas, these approaches, while being close to quantitative in terms of efficiency at a collection level, are hardly applicable when extraction kinetics is under consideration. Kinetics of SFE is always studied whenever an economically effective extraction method, be that analytical or preparative, is designed. Such research is of particular value for industrial-scale applications of SFE, where knowledge of extraction kinetics in terms of total mass yield versus time is imperative for process modeling and scale-up [12,38,39,40,41]. Experimentally built extraction kinetic curves are required to perform modeling. Neither solid nor liquid trappings allow accurate repetitive weighing of collected mass during the extraction process. An error introduced by the inexactitude of mass measurement of a solid or a liquid trap operated in the stream of rapidly blowing gaseous CO_2_ is larger than the mass difference arising from sample collection. Thus, only dry collection and weighing of a free sample can be applied for building total mass kinetic extraction curves in SFE.

Sampling during kinetic curve building can be performed using a piece of tubing with a restrictor on its end and exchangeable glass vials. The tip of the tube coming out from the extractor backpressure regulator is put into the vial, which collects the extract. The vial is replaced by a new one in due time, and when the process is complete, all the vials are weighed and thus a kinetic curve is built. The approach is simple and convenient, but it suffers from serious mass losses, both because of poor entrapment of volatiles and unstable operation of the restrictor. A cold aerosol moving with high linear velocity after the backpressure regulator is difficult to operate with. Volatile components tend to get blown away by it and not to condense in the vial. Dry ice tends to form due to the Joule-Thomson cooling, which blocks tubing and creates unwanted pressure increase in the collection unit. After such blockage gets pushed through by upcoming portions of the fluid, a released stream causes perturbations in the vial, which leads to further losses of the collected material. It affects the accuracy of the extraction method development and scale-up. Still, the alluring simplicity of the tubing-and-vial approach makes it a widespread choice in SFE research. Another alternative is a relatively large volume pressure-controlled heated cyclone separator, which is good for raw materials containing large amounts of non-volatile extractables, such as seeds or nuts, but is poorly suited for aroma plants as they usually contain much smaller quantities of extractable material, which is more prone to evaporation than vegetable oil. Several groups have designed their special instruments to cope with these problems [42,43]. Recently, we proposed a design for a simple low-pressure 3D-printed cyclone separator for SFE, especially developed to tackle the above-mentioned issues [44]. Here, we demonstrate its applicability to a more accurate kinetic curve building in SFE. The aim of the work was to compare the newly designed device to a standard tubing-and-vial routine in SFE from aroma plants and to find out whether or not improvement in collection is significant for extraction kinetics research.

## 2. Results and Discussion

Two types of aroma plants were used as model raw materials: laurel (*Laurus nobilis* L.) and rosemary (*Rosmarinus officinalis* L.). These plants were chosen because they contain substantial amounts of essential oil, for which the problem of collection in SFE is relevant, and also because both these plants give reasonable yet not large mass yield in SFE at high extraction pressures (ca. 3–5%) [42,45,46], which makes them representative testing objects for kinetic curve building.

**Figure 1:**
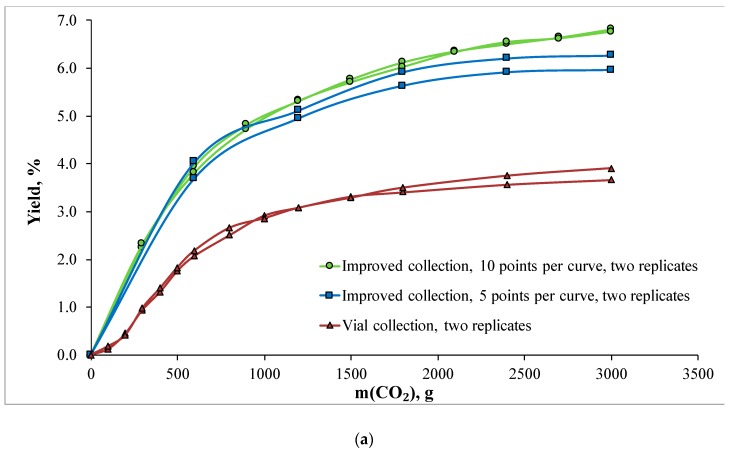

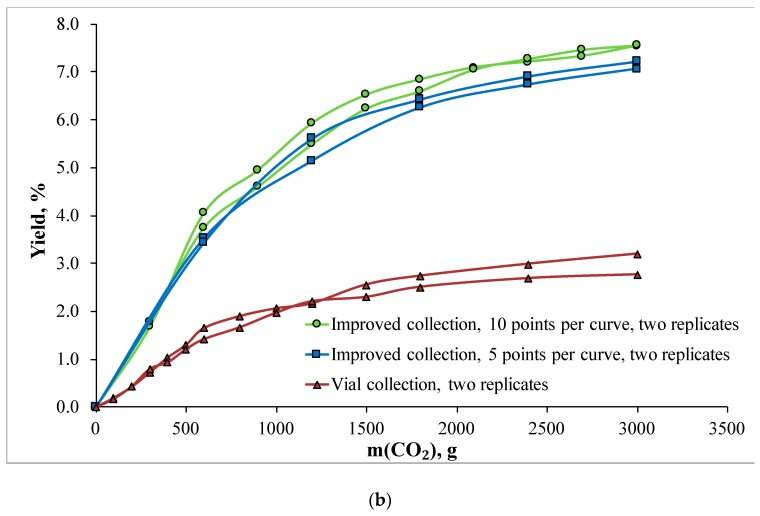
Supercritical fluid extraction kinetic curves for (**a**) laurel and (**b**) rosemary. Green circles: enhanced collection device, 10 points of mass measurement per curve. Blue squares: enhanced collection device, 5 points of mass measurement per curve. Red triangles: direct collection into vials. The curves are intentionally given as spline interpolations instead of approximation so that break points are visible. Two replicates are shown for each set of collection condition to demonstrate qualitative reproducibility of the results.

Two methods of sample collection were compared in this work: (1) direct collection into vials using a piece of tubing with a restrictor on its end and (2) collection using an enhanced collection device showing better recovery, which was designed in our lab [44]. Representative examples of the extraction kinetic curves built using these approaches are shown in Figure 1.

The most important observation made from the presented results is a substantial difference in extraction mass yield between the two collection methods. Its magnitude is striking: overall yield for laurel was 6.8% for the enhanced collection device against 3.8% for tubing-and-vial collection. The difference was even bigger for rosemary: 7.5% when enhanced collection device was used versus 3.0% when tubing-and-vial method was applied. Moisture analysis of raw materials before and after extraction showed that this difference could not be attributed to the difference in water content. Experiments were performed at the same air humidity values, and measures were taken to avoid condensation of water vapors from air into collection vials. Visually, obtained extracts looked the same for two collection methods. Results showed reasonable quantitative reproducibility: the presented yield values were reproduced at least twice within ± 0.2 g range for every experiment except the vial collection for rosemary, for which the range was ± 0.3 g. Mass yields observed for the enhanced collection device were rather close to the yield values calculated from the raw material mass loss after extraction. The latter were ca. 9–11% for laurel and ca. 8–9% for rosemary. Note that these values are inevitably oversized as raw material mass loss measurement requires taking extracted material out of the vessel, which cannot be performed with 100% accuracy for obvious mechanical reasons.

We attribute the observed difference to a better collection of volatile components by the enhanced collection device. Cyclone separation and spray trapping allow the carryover of droplet in the form of aerosol to be avoided, whereas proper check valve heating prevents dry ice formation and simultaneously provides enough cooling for effective condensation of volatiles. For this work, the value of 150 °C was found to be an optimum temperature of the check valve heating bath (see Figure 3b and Section 3.3 for details). Preliminary experiments with higher temperatures (200 °C) showed mass yields lower by 1–2 percentage points. As volatiles often represent the most valuable part of aroma plant extracts, their accurate collection during SFE kinetics investigation is of crucial importance, especially when these data are planned to be used to design a preparative-scale process.

It should be stated that the extraction conditions applied here were not meant to be optimal for the two chosen model aroma plants in regards of extract composition. Typically, the SFE of aroma plants is performed using lower pressure values, and extracts are usually fractionated during collection via step-wise pressure decrease and winterization of non-volatiles in the first separator [42]. However, that was not the aim of this work. Instead, we used higher extraction pressure and collected extracts without fractionation in order to increase the yield and thus emphasize the difference between two collection approaches.

Apart from providing more accurate sample collection, the enhanced device also allows the use of much larger flow rates, thus drastically decreasing experiment time. The current design of the device allows the use of flow rates up to 30 g/min, whereas with vials, we could not work at flow rates higher than 10 g/min without the risk of dry ice clogging. Typically, SFE kinetics research is conducted at even lower flow rates [42,47,48] to avoid the collection problems described above. This means that building one kinetic curve usually requires a full working day. With our enhanced collection device, at least a three-fold increase in data gain productivity can be achieved.

A curious effect was noticed when different numbers of mass measurement points per curve were tested. An increase in the number of points per curve led to an apparent increase in extraction mass yield (see green and blue lines in Figure 1a,b). The magnitude of the effect was modest but not negligible: for laurel, 10 points per curve gave yield value of 6.8%, whereas 5 points per curve gave 6.1%; for rosemary, 10 points per curve resulted in 7.5% yield, whereas 5 points per curve gave 7.1%. We attribute this to the effect of the static–dynamic extraction regime, which was realized unintentionally when using our collection device. To measure the mass of a fraction, we stopped CO_2_ flow, detached the cyclone separator alone with the collection vial from the instrument, and weighed it. This device is quick-detachable, and the whole weighing procedure does not require more than 1 min. However, judging from the comparison data given in Figure 1, it was enough to cause additional increase in mass yield. Noticeably, the kinetic curves with 10 and 5 points per curve virtually coincided at the first, linear interval and started differing only from the break point between the solubility-limited and diffusion-limited extraction stages. In our case, this point was around 600 g of CO_2_ for both raw materials. This is in good accordance with the hypothesis of the static–dynamic regime effect. Such extraction regimes have been thoroughly investigated in the past, and it is known that the addition of a static regime improves extraction kinetics at the diffusion-limited stage. The first stage of the extraction is limited primarily by the solubility of an extract in supercritical CO_2_, and due to the high dissolution rates in supercritical fluids, adding a static step at this stage has little effect on yield. In contrast, the second stage is diffusion-limited, and putting static steps there gives the extractable components additional time to diffuse from the particle core to the surface. As static–dynamic regimes are seldom used on a preparative scale nowadays, this effect may give a slight yield overestimation. In order to avoid that, one might use fewer mass measurement points at the second part of the kinetic curve. A practical recommendation could be to use more points at the beginning of the curve in order to locate the curve break point more accurately and then to diminish the number of points in order to make the kinetic data closer to the ones that would be observed during preparative extraction.

Thus, the new collection device designed for SFE showed better extract trapping and can be recommended for experiments where accurate mass yield measurement is required.

## 3. Materials and Methods

### 3.1. Materials

Fresh laurel (*Laurus nobilis* L.) leaves were collected by S.A. Bagatelia (Sukhum Physical-Technical University, Sukhum) in September 2019 and then air-dried in our lab for several weeks. Rosemary leaves were purchased from a local food store in Moscow and used as such without drying. Laurel leaves were first subjected to coarse grinding in a blender for 1 min and then to milling in a two-blade mill for 1 min. Rosemary leaves were milled directly in a two-blade mill without preliminary coarse grinding. The milled material was sieved through a 1 mm sieve. Material milling and sieving was performed right before the extraction. A portion of the prepared material was taken for humidity measurement, which was performed using a Mettler Toledo MJ-33 moisture analyzer.

Food-grade CO_2_ (99.8%) was supplied by Linde Gas Rus (Balashikha, Russia).

Amber glass 60 mL vials were purchased from Merck via Galachem (Moscow, Russia).

Polytetrafluoroethylene (PTFE) rods of 10 mm width were purchased from a local store. Preliminary testing showed no extraction from this Teflon material in the conditions applied in this work. No extractables and no weight change was observed after supercritical extraction of pieces cut from these rods.

### 3.2. Instrumentation

**Figure 2:**
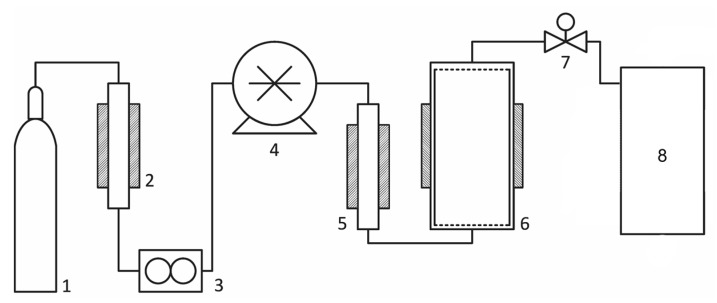
Schematic diagram of SFE-1000 extraction system. 1: CO_2_ cylinder, 2: cooling heat exchanger, 3: Koriolis flowmeter, 4: two-piston high-pressure pump, 5: electrical heater, 6: 1 L extraction vessel with an electrical heating jacket, 7: manual backpressure regulator, 8: collection system.

All extraction experiments were performed using Water Corp SFE-1000 extraction system. A schematic diagram of the system is depicted in Figure 2.

**Figure 3:**
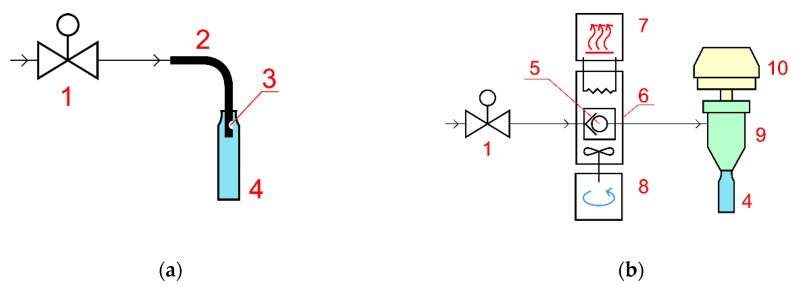
Schematic diagrams of vial collection (**a**) and enhanced collection (**b**) units. 1: backpressure regulator, 2: stainless steel 1/8′’ OD tubing, 3: restrictor, 4: glass vials, 5: check valve, 6: heating bath, 7: heating control module, 8: mixer, 9: cyclone separator, 10: spray trap.

Two types of collection systems were compared: a standard tubing-and-vial collection and our specially designed collection system. Schematics of the two collection units are depicted in Figure 3.

In the case of vial collection (Figure 3a), outlet flow from the backpressure regulator followed through the steel tubing with the restrictor on its end into a 60 mL vial and expanded right inside it. The tubing was immersed into the vial to a depth of 10 mm. Vials were changed manually at certain intervals according to the experimental procedure.

The collection system depicted in Figure 3b consists of a cyclone separator body, a spray trap, and a heated check valve. An outlet flow from the extraction system backpressure regulator goes to a check valve. The purpose of the valve is to keep the fluid at elevated pressure so that the extract is sustained in solution and does not start precipitating from CO_2_ before it reaches the cyclone separator. The check valve is a one-way valve with a spring opening at roughly 80 bar. Right after the valve pressure is released. The valve is immersed into a controllable heating bath in order to adjust expanding fluid temperature. The heating bath consists of an electrical heater, liquid heat carrier, impeller, and a control box. If no heating is applied, then dry ice forms and blocks the valve and the tubing, which ruins collection. If heating is too strong, then volatile components will not get condensed within the cyclone separator but rather be blown away by the outlet flow of gaseous CO_2_. The temperature should be adjusted in such a manner that no dry ice clogging occurs but the cooling effect is still present in order to enhance trapping of volatiles. The required heating level depends on extraction pressure, temperature, CO_2_ flow rate and, to a certain extent, on extract composition; it should be adjusted for a particular case. In this work, the heating bath working temperature was set to 150 °C in all experiments. Polydimethylsiloxane with a viscosity of 50 cP served as the heat carrier. After the valve, the flow gets directly into the cyclone separator, where gaseous CO_2_ gets separated from the extract. The top outlet of the separator is equipped with a spray trap to prevent small droplets of extract from being carried out of the system by CO_2_. Collecting vials are screwed to the bottom of the separator body, and the extract is collected in them. The separator is quick-detachable from the check valve unit. Both the separator and the spray trap are made from PET-G using 3D printing (fused filament fabrication), which makes them light and easily adjustable. A detailed description of all the units of this collection system along with full open-source technology for its manufacturing is being published elsewhere [49]. During kinetic curve building, the vial is not detached from the separator; instead the whole separator-spray trap-vial assembly is detached, weighed, and reattached. For this, CO_2_ flow in the extraction system is temporarily stopped for about 1 min.

### 3.3. Supercritical Fluid Extraction Procedure

An amount of 100 g of freshly prepared raw material was placed into a 1 L extraction vessel. It occupied about a third of the vessel volume. The rest of the volume was filled with 10 mm long PTFE beads cut from a 10 mm width rod. The vessel was sealed and heated up to 40 °C. After temperature equilibration, CO_2_ flow was started. The initial pressure build-up within the extraction system was performed at 50 g/min flow rate. Upon reaching the required extraction pressure, the flow rate was reduced to 30 g/min in case of our collection system and to 10 g/min in case of vial collection. Larger flow rate was unachievable for the tubing-and-vial method because vials got completely blocked by the forming dry ice. Extraction was carried out at a pressure of 300 bar. For vials, mass measurements were performed every 10 min for the first hour, every 20 min for the second hour, every 30 min for the third hour, and then every 60 min till the end of the experiment. For enhanced collection system, mass measurements were performed every 10 min throughout the whole run. The total extraction time was 300 min for the tubing-and-vial method and 100 min for the enhanced collection system. In both cases, the overall mass of pumped CO_2_ was 3 kg, which gave solvent-to-feed ratio equal to 30. All experiments were repeated at least twice. After the run, the system was depressurized, the residual raw material was taken out for weighing, the collection system was disassembled, and wetted surfaces of the equipment were thoroughly rinsed and dried to avoid cross-contamination between runs.
